# Hepatitis C micro-elimination through the retrieval strategy of patients lost to follow-up

**DOI:** 10.1186/s12876-023-02665-y

**Published:** 2023-02-13

**Authors:** Cheng-Jen Chen, Yung-Hsin Huang, Chao-Wei Hsu, Yi-Cheng Chen, Ming-Ling Chang, Chun-Yen Lin, Yi-Hsien Shen, Rong-Nan Chien

**Affiliations:** 1 Division of Hepato-Gastroenterology, Lin Kou Chang Gung Memorial Hospital, Taoyuan, Taiwan, ROC; 2 Chang Gung University College of Medicine, Taoyuan, Taiwan, ROC

**Keywords:** Hepatitis C, Micro-elimination, Direct-acting antivirals, Retrieval strategy

## Abstract

**Background and aim:**

World Health Organization sets up an ambitious and attainable goal to eliminate hepatitis C (HCV) by 2030. The previous diagnosed HCV patients lost to follow-up were considered as an important target group for HCV elimination. We conducted a call back program to retrieve the lost to follow-up HCV patients and link them to care in our hospital. By analyzing and comparing our result with that from other studies, we wish to improve our retrieval strategy and provide our experience to the general communities.

**Methods:**

A list of the patients with a medical record showing seropositive for antibody to HCV (anti-HCV Ab) from 2004 to 2017 was retrieved by the department of intelligent technology of our hospital. Three dedicated staff members reviewed the patients’ electronic medical records (EMRs) and recruited the patient lost follow-up to the call back program. The staff members contacted the qualified patients by telephone and inquired about their opinions for treating their chronic HCV infection. We also informed the patients about the retrieval strategy and why we contact them. As our National Health Insurance request, we gave all patient one **i**nformed consent for hepatitis C treatment. Informed consents have been obtained from all patients. Referrals to our gastroenterology unit (GU) were arranged for the patients who would like to continue their chronic HCV care in our hospital.

**Results:**

There were 31,275 anti-HCV positive patients. We included 11,934 patients (38.2%) into the call back system and contacted them by telephone. Based on the response to our call, we ascertained 1277 eligible cases (10.7%) for retrieval. The patients who were younger (< 55), lived in Taoyaun City or had tested positive for anti-HCV Ab at the department of internal medicine department had an increased rate of successful call back. There were 563 patients (44.1%) returning to our GU. Of them, 354 patients (62.9%) were positive for HCV viremia. 323 patients (91.2%) received the DAAs treatment. The SVR12 with Grazoprevir + elbasvir, Glecaprevir + pibrentasvir, Sofosbuvir + ledipasvir and Sofosbuvir + velpatasvir were 97.9%, 98.8%, 100% and 97.5%, respectively.

**Conclusions:**

Call back system can expand our reach to those unaware or ignoring chronic HCV infection patients and link them to treatment.

## Introduction

Chronic hepatitis C virus (HCV) infection poses an ongoing threat to public health [1]. Chronic HCV infection leads to the development of cirrhosis in approximately 10–20% patient over 20–30 years. Once cirrhosis is established, there is an annual 1–5% risk of hepatocellular carcinoma and an annual 3–6% risk of hepatic decompensation [2]. The global prevalence is estimated to be 1.0%, which corresponds to 71.1 million infected patients [3]. In Taiwan, the estimated prevalence rate increases to 3.28% [4] and there are approximately 4 hundred thousand infected patients [5].

The primary goal of HCV therapy is to cure the infection and to achieve sustained virological response (SVR), defined as undetectable HCV RNA after treatment completion [6]. Traditionally, the initial HCV therapies are IFN based regimens which are poorly tolerated, associated with severe adverse effects and result in SVR between 40 and 65%. After the introduction of the direct-acting antivirals (DAAs), SVR improves significantly without serious adverse effects or complications. Therefore, World Health Organization (WHO) sets up an ambitious and attainable goal to eliminate HCV by 2030 [7].

Due to the scale and complexity of HCV elimination, European Association for the Study of the Liver (EASL) proposes a “micro-elimination” approach which focuses and treats different populations and at-risk groups. Ultimately, micro-elimination will lead to macro-elimination [8, 9]. Other than the initial recognized target groups of micro-elimination, such as prisoners [10], children and/or hemophilia patients, the previous diagnosed HCV patients lost to follow-up were also considered as an important target group for HCV micro-elimination [11–13].

In Taiwan, DAAs have been reimbursed since 2017 and are currently available for HCV treatment regardless of liver fibrosis status [4]. Our government has claimed to eliminate HCV by 2025, 5 years prior to the mission by World Health Organization and aims to treat two hundred fifty thousand patients by 2025. In order to fulfill this goal, the ministry of health and welfare sets up 3 policy directions, namely (1) therapy spearheads prevention, (2) screening supports therapy and (3) prevention secures outcome [5]. Retrieving the lost to follow-up HCV patients is an important method to increase the therapy coverage. The hospitals are encouraged to organize call back programs to retrieve the HCV patients lost to follow-up for further evaluation and subsequent treatment [14].

In this study, we conducted a call back program to retrieve the lost to follow-up HCV patients and link them to care in our hospital. By analyzing and comparing our result with that from other studies, we wish to improve our retrieval strategy and provide our experience to the general communities.

## Materials and methods

Chang Gung Memorial Hospital, Lin Kou branch, is the only medical center in Taoyuan City, within Northern Taiwan. With annual 4 million outpatient visits, the hospital provides health care not only for the patients from Taoyuan City, but also for those from other cities of Taiwan.

A list of the patients with a medical record showing seropositive for anti-HCV Ab from 2004 to 2017 was retrieved by the department of intelligent technology of our hospital. Three dedicated staff members from our hepatic diseases center reviewed the patients’ electronic medical records (EMRs) to evaluate the patients’ current status and recruited the patient lost follow-up to the call back program. The patients with the following conditions were excluded: (1) below age 11 and above age 80, (2) poor medical status (Poor medical status means that the patients have the following conditions: (i) Patients got some kind of diseases which would make them hard to return to our clinic for follow up such as severe heart or pulmonary disease (For example, patients lived in respiratory care ward). (ii) Some patients got advanced stage or even terminal stage of cancers with poor prognosis. Curing hepatitis C would not improve quality of their life, so we did not include these patients in our study. (iii) Some patients already moved to somewhere far away from our hospital.), (3) false positive for anti-HCV Ab, (4) documented negative HCV-RNA record, (5) without valid telephone numbers, (6) complete DAA treatment with SVR, and (7) in the care of a gastroenterologist or general surgery. The staff members contacted the qualified patients by telephone or mails since April 2019 till October 2020 and inquired about their opinions for monitoring and/or treating their chronic HCV infection. Referrals to our gastroenterology unit (GU) were arranged for the patients who would like to continue their chronic HCV care in our hospital. A full-time fellow was appointed to analysis the result of the call back program.

### Statistical analysis

Continuous variables were expressed as n/N (%), mean and standard deviation. Categorical variables were expressed as numbers and percentages. One-sample Kolmogorov–Simirnov test was used to evaluate the distribution of continuous variables. Student’s t-test was used to compare continuous variables whereas Chi-square test was used to compare categorical variables. All statistical data were analyzed using the SPSS software version 23.0 (SPSS Inc., Chicago, IL, USA). A *P* < 0.05 was considered significant.

### Ethical considerations

This study was approved by the Chang Gung Medical Foundation Institutional Review Board (IRB No. 202101165B0).

## Result

**Fig. 1:**
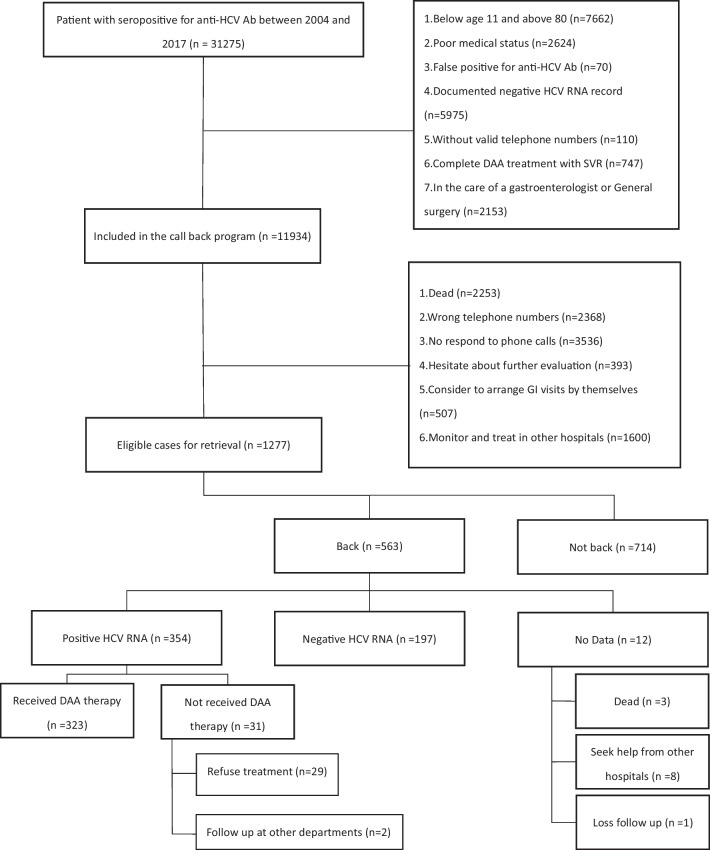
Flow chart of the retrieval strategy

**Fig. 2:**
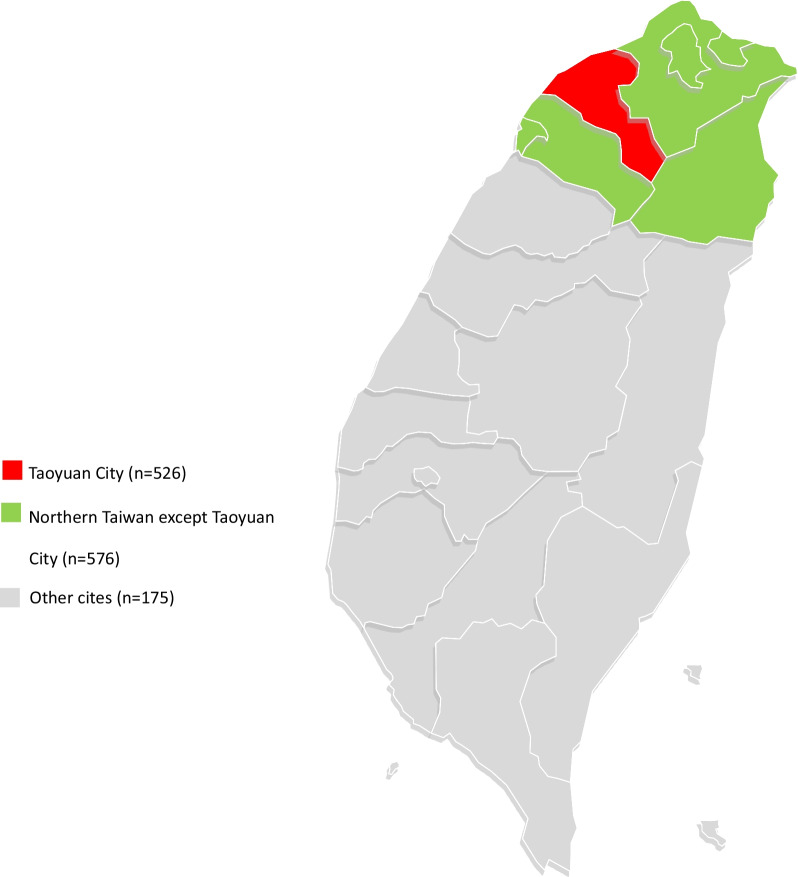
Distribution of the eligible cases for retrieval

In a period of 14 years, there were 31,275 anti-HCV positive patients. We included 11,934 patients (38.2%) into the call back system and contacted them by telephone since April 2019 till October 2020. Based on the response to our call, we ascertained 1277 eligible cases (10.7%) for retrieval. Figure 1 illustrates the study’s enrollment flow chart. We analyzed the 1277 eligible cases and summarized their demographic characteristics in Table 1. The majority of patients was within the 55- to 70-year-old group. The patients who were younger (< 55), lived in Taoyaun City or had tested positive for anti-HCV Ab at the department of internal medicine department had an increased rate of successful call back. Whereas, the patients who were older (> 70), lived in Northern Taiwan (except Taoyuan City) and other cities in Taiwan, and had tested positive for anti-HCV Ab at the department of surgery had a decreased rate of successful call back. Figure 2 shows geographic locations of Taoyuan City, Northern Taiwan and other cities.

**Table 1:** Baseline demographics between call back and not back patients

	All patient (1277)	Back (563)	Not back (714)	*p* value
Age, year, mean	60.37 ± 12.00	58.58 ± 11.74	61.79 ± 12.02	0.000
Age level, n/N (%)				
< 55	369/1277 (28.9%)	187/56 (33.2%)	182/714 (25.5%)	0.003
55–70	628/1277 (49.2%)	288/563 (51.2%)	340/714 (47.6%)	0.231
≥ 70	280/1277 (21.9%)	88/563 (15.6%)	192/714 (26.9%)	0.000
Gender (M), n/N (%)	635/1277 (49.7%)	295/563 (52.4%)	340/714 (47.6%)	0.101
Residence, n/N (%)				
Taoyuan City	526/1277 (41.2%)	289/563 (51.3%)	237/714 (33.2%)	0.000
Northern Taiwan (except Taoyuan City)	576/1277 (45.1%)	235/563 (41.7%)	341/714 (47.8%)	0.028
Other cities	175/1277 (13.7%)	39/563 (6.9%)	136/714 (19.0%)	0.000
Physicians/N (%)				
Internists	748/1277 (58.6%)	375/563 (66.5%)	373/714 (52.2%)	0.000
Non-internists	407/1277 (31.8%)	143/563 (25.4%)	264/714 (37.0%)	0.000
Health check-up	122/1277 (9.6%)	46/563 (8.1%)	76/714 (10.6%)	0.131

There were 563 patients (44.1%) returning to our GU. Of them, 354 patients (62.9%) were positive for HCV viremia. After discussion with doctors, 323 patients (91.2%) received one of the 4 DAAs (elbasvir/grazoprevir, sofosbuvir/ledipasvir, glecaprevir/pibrentasvir, sofosbuvir/velpatasvir) therapy. (The selection criteria of DAA were according available data of hepatitis C treatment at that time. We would select proper DAA according to the genotypes of hepatitis C, treatment history of hepatitis C before, and the drugs history of patients. All DAA choices were made after discussion between physician and patients. How to avoid drug-drug interaction was one important issue when we selected regimens). The overall sustained virological response rate at off-treatment 12 weeks (SVR12) was 98.1%. There was no significant relationship between the DAAs and SVR12 rate. Table 2 provides basic information of the patients receiving DAA treatment in the call back program.

**Table 2:** Patient characteristics between different DAA according to different genotype

	All (N = 323)	Grazoprevir + elbasvir (Zepatier) (N = 48)(14.9%)	Glecaprevir + pibrentasvir (Maviret) (N = 83)(25.7%)	Sofosbuvir + ledipasvir (Harvoni) (N = 32)(9.9%)	Sofosbuvir + velpatasvir (Epclusa) (N = 160)(49.5%)	*P* value
Male	162 (50.5%)	16 (33.3%)	52 (62.7%)	15 (46.9%)	79 (50.0%)	0.013
Age (years)	59.0 ± 11.28	61.02 ± 10.93	57.87 ± 11.92	55.81 ± 12.82	59.62 ± 10.62	0.144
Genotype 1a	28(8.7%)	0 (0%)	11 (13.3%)	2 (6.3%)	15 (9.4%)	0.069
Genotype 1b	131(40.6%)	48 (100%)	21 (25.3%)	*8 (25.0%)*	54 (33.8%)	0.000
Genotype 2	133(41.2%)	0 (0%)	42 (50.6%)	19 (59.4%)	72 (45.0%)	0.000
Genotype 3	3(0.9%)	0 (0%)	0 (0%)	*0 (0%)*	3 (100%)	0.379
Genotype 6	18(5.6%)	0 (0%)	6 (7.2%)	3 (9.4%)	9 (5.6%)	0.246
Genotype Mix	10(3.1%)	0 (0%)	3 (3.6%)	*0 (0%)*	7 (4.4%)	0.32
RNA (IU/mL)(10^6^)	4.39 ± 5.96	3.04 ± 4.82	4.89 ± 6.6	2.93 ± 3.63	4.78 ± 6.24	0.098
INR	1.08 ± 0.11	1.09 ± 0.21	1.09 ± 0.07	1.1 ± 0.08	1.07 ± 0.08	0.447
WBC (x/μL)(10^3^)	6.15 ± 1.97	6.18 ± 1.97	6.16 ± 1.80	5.88 ± 1.81	6.20 ± 2.09	0.879
Hb (g/dL)	13.66 ± 2.06	13.30 ± 1.71	13.32 ± 2.38	13.85 ± 1.67	13.93 ± 2.01	0.075
Platelet (x/μL)(10^3^)	206.95 ± 68.52	213.18 ± 70.62	200.29 ± 66.58	196.90 ± 75.67	210.58 ± 67.65	0.500
Albumin (g/dL)	4.3 ± 0.39	4.33 ± 0.29	4.23 ± 0.44	4.4 ± 0.28	4.30 ± 0.41	0.163
AST (IU/L)	53.55 ± 45.91	43.71 ± 27.71	50.47 ± 41.26	50.44 ± 37.64	58.76 ± 53.18	0.189
ALT (IU/L)	64.13 ± 67.22	47.15 ± 40.77	64.34 ± 66.76	65.22 ± 70.36	68.92 ± 72.73	0.275
T-Bilirubin (mg/dL)	0.72 ± 0.41	0.63 ± 0.3	0.68 ± 0.37	0.81 ± 0.47	0.74 ± 0.44	0.162
AFP (ng/mL)	21.26 ± 156.39	12.36 ± 55.86	5.19 ± 7.37	6.75 ± 9.40	35.52 ± 220.73	0.438
Fibrosis F3	42 (13.0%)	11 (22.9%)	9 (10.8%)	3 (9.4%)	19 (11.9%)	0.170
Fibrosis F4	37(11.5%)	2 (4.2%)	15 (18.1%)	3 (9.4%)	17 (10.7%)	0.098
EOT	321 (99.4%)	47 (97.9%)	83 (100%)	32 (100%)	159 (99.4%)	0.496
SVR12	317 (98.1%)	4 7(97.9%)	82 (98.8%)	32 (100%)	156 (97.5%)	0.759

EOT, end of treatment; SVR12, sustained virological response at week 12

**Fig. 3:**
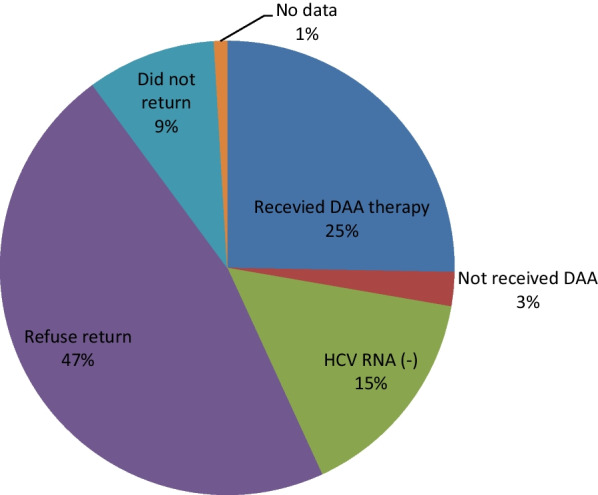
Outcome of call back result

In this study, we called back total 1277 cases. Figure 3 showed 25% of patients received DAA therapy. 47% of patients refused appointments. 9% of patients did not return to appointments. 15% of patient showed HCV RNA negative. 1% of patient did not complete the exam after appointments. 3% of patients did not received DAA therapy (refused treatment after appointments or follow up at other departments).

**Fig. 4:**
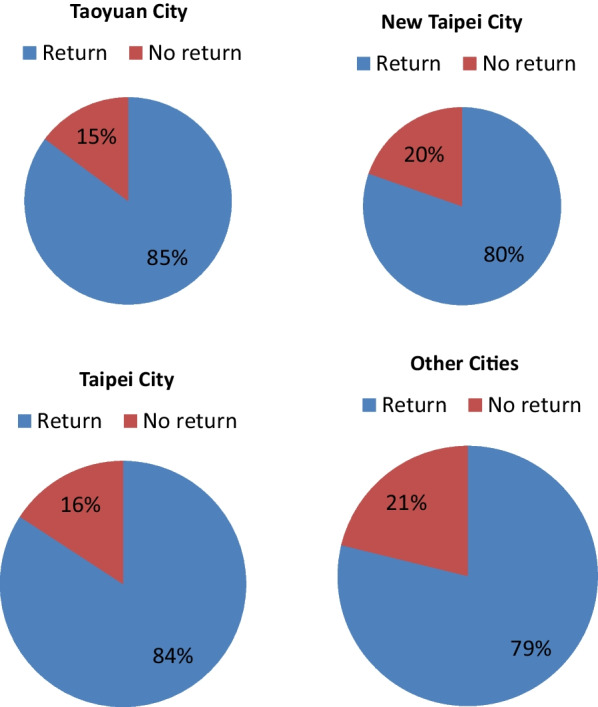
Compare return rates

We compared the return rates in Fig. 4 and found that the patients living in Taoyaun City were more willing to return to our hospital than the patients in Northern Taiwan (except Taoyuan City) and other cities in Taiwan with the return rate up to 85%.

**Fig. 5:**
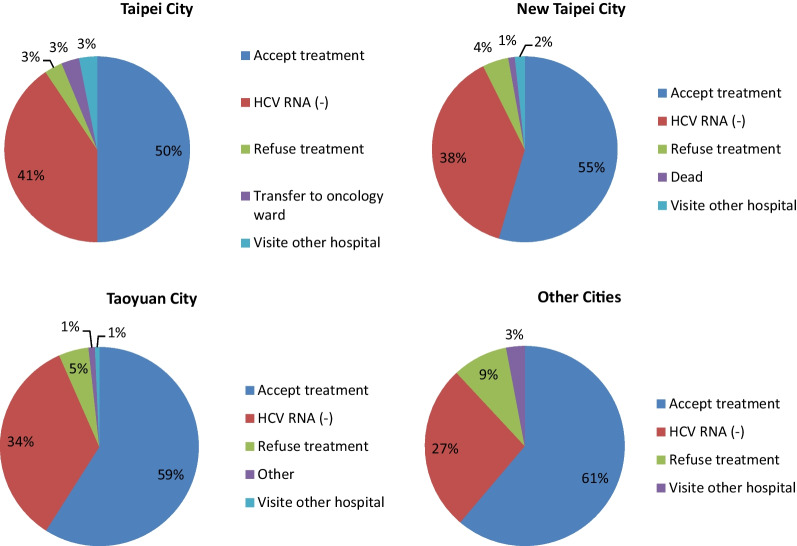
Compare outcome of return

We further analyzed the call back outcome in different cities (Fig. 5). The refuse treatment rate was highest in other cities (9%) and lowest in Taipei city. Taipei City has the lowest proportion of patients receiving treatment but the highest proportion of patients with HCV RNA (−). In contrast, other cities have the highest proportion of patients receiving treatment but the lowest proportion of patients with HCV RNA (−).

## Discussion

This retrospective one-center study reported a result of micro-elimination on retrieving the HCV patients lost to follow-up for further evaluation and management. Through the call back program, we ascertained 1277 eligible patients (10.7%). Among these patients, 564 patients (44.1%) were retrieved successfully and returned to the GU. 359 patients (62.9%) were positive of HCV viremia and 323 patients (91.2%) received DAAs. The overall SVR12 rate was 98.1%

The number of the eligible patients was considerably lower than that of the included patients in the call back program and a huge proportion of the patients were not reached either due to wrong telephone numbers or no responding to phone calls up to total 5904 cases (49.5%). The primary reason was the lacking of the updated contact information. Because of the Taiwan privacy regulation, we could only utilize the patients’ contact information recorded in their EMRs and not able to use other databases, such as the household registration system, to search for updated contact details. One retrieval program in Holland encountered the same limitation [15]. We also lost of accessibility to patients after they moved out or dead.

After analyzing the demographic characteristics of the eligible patients, we figured out three factors which may influence the patient’s decision on retrieval. Those factors were the patient’s age, the physicians who ordered HCV testing and the patient’s residence. In this study, we found the patients who were younger (< 55), lived in Taoyaun City or had tested positive for anti-HCV Ab at the department of internal medicine department had an increased rate of successful call back.

A study focusing on baby boomers in US revealed that age was a predictor of knowledge about HCV and there was a decrease in knowledge for every year increase in age [16]. Lack of knowledge about HCV had been identified as a barrier for patients seeking help [17]. These evidences could explain why the younger patients in our study were more willing to return to the GU for further evaluation. The same phenomenon was also observed in another retrieval program in Taiwan [14].

The physician’s perception towards HCV was quite different between the internists and the non-internists. The internists often ordered HCV testing to survey the cause of abnormal liver function tests, whereas the non-internists often ordered the test for pre-operative evaluation. In one study evaluating the intra-hospital HCV patient referral rate, the internists referred more patient to the hepatologist that the non-internists did [18]. In our study, the patients diagnosed by the internists showed an increased retrieval rate, compared with the patients diagnosed by the non-internists. It was logical to deduce that physician’s perception of HCV [19] might also influence his patient’s awareness of the disease and his response on receiving our telephone call. In our study, 47% of patient directed refuse return after receiving phone call. This might due to lack of knowledge about HCV and did not care about their health problem. HCV-related education seems to be also important to improve with prevention and elimination of HCV [20]. Efforts to link patients back to HCV care with early and easy access to HCV treatment are necessary to reach the HCV elimination goal [21].

The patient’s residence also influenced his decision on returning to the hospitals for further evaluation [22]. One study in England revealed that the treated HCV patients lived closer to the hospitals than the patients lost to follow-up did and living within 4 km of a treatment hospital was a strong indicator of having started treatment [23]. In line with that study, our study showed that the patients living in Taoyaun City were more willing to return to our hospital than the patients in Northern Taiwan (except Taoyuan City) and other cities in Taiwan.

In our study, 91.2% of the retrieved HCV viremia patients received DAAs treatment. Genotype 2 was the major genotype (41.2%), followed by genotype 1b (40.6%). Treatment regimen was based on genotype and clinical practice. The SVR12 rates of patients treated with Grazoprevir + elbasvir, Glecaprevir + pibrentasvir, Sofosbuvir + ledipasvir and Sofosbuvir + velpatasvir were 97.9%, 98.8%, 100% and 97.5%, respectively (Table 2). Throughout the treatment course, no severe adverse effects occurred. In line with other real-world studies in Taiwan, the DAAs were effective and well tolerated [24, 25].

We also found the socioeconomic status or the development level of cities might also influence patient’s diseases status and attitude to receiving treatment. In our further subgroup analysis, the refuse treatment rates were opposite to socioeconomic status. The rates of HCV (−) increased according to socioeconomic status. Same situation was also noted in Danish [26, 27]

Our study has several limitations. First, we could not recruit more eligible patients into the call back program due to the lacking of the updated contact information. Second, we identified three factors which might influence the retrieval rate. However, due to the retrospective nature of the study, we could not design and perform a questionnaire to confirm our hypothesis.

In this study, 25% of retrieval patients received DAA therapy and 15% of patients showed HCV RNA (−). The call back system really can improve HCV micro-elimination. However, we also found 47% patients refused to return at begin.

How to change the cognition of patients who refused to return is the key to improve the efficiency of call back system. This requires more publicity and education from hospitals and governments. How to retain or update patients’ contact information is also very important. This may be improved by periodically reminding from computer system to update the patients’ contact information.

The proportion of patients that refused therapy was small (8.2%). This represented most patient could understand and willing to receive treatment. Most patients refused treatment due to they received other therapy at that time. (Some patients informed us that they wanted to receive chemotherapy about their cancer first.) Some patients still did not think that it is important to cure hepatitis C due to they did not feel uncomfortable. We could overcome this challenge with patience and health education. If patient really needed to complete some treatment first, we could follow up their condition at OPD and arrange hepatitis C treatment later.

In 2018, our government has claimed the target to eliminate HCV by 2025. The government has prepared a large budget on treatment and propagates to achieve the goal. We also keep on calling back patients and improving our strategy after this study.

## Conclusion

Call back system can expand our reach to those unaware or ignoring chronic HCV infection patients and link them to treatment resulting in improving the goal of HCV micro-elimination. Patient’s age, the physicians who ordered HCV testing and the patient’s residence may influence the patient’s decision on retrieval. In the hospital care setting, DAAs are safe and effective with high SVR12.

## Data Availability

The datasets used during the current study available from the corresponding author on reasonable request.
